# Battle of the Genomes: The Struggle for Survival in a Microbial World

**DOI:** 10.3201/eid1306.070332

**Published:** 2007-06

**Authors:** Marta Gwinn

**Affiliations:** * Centers for Disease Control and Prevention, Atlanta, Georgia, USA

Although this book’s title promises the excitement of a 21st-century computer game, the cover photograph of Robert Koch in 1883 provides a better clue to the contents. The general plan is a survey of 20th-century genetics, illustrated by insights into human coevolution with microbial pathogens. Early chapters focus on familiar examples, including G6PD deficiency and sickle cell trait as adaptations to malaria, as evidence for pathogen-driven natural selection. Later chapters discuss more recent research findings, varying from female preference for the scent of males with dissimilar human leukocyte antigen types to the role of human CFTR membrane protein in infection with *Salmonella* Typhi. All of these are such good stories that science writer Matt Ridley included briefer versions in Chapter 9 of his popular book Genome: The Autobiography of a Species in 23 Chapters (*1*).

Battle of the Genomes: The Struggle for Survival in a Microbial World discusses in some detail how catastrophic epidemics of cholera, bubonic plague, and smallpox could explain the emergence of certain common human genetic mutations. Some of these mutations are deleterious; for example, CFTR ΔF508, which reduces the risk for typhoid, causes cystic fibrosis in persons who inherit 2 copies. Other mutations are beneficial, such as CCR5 Δ32*,* which may have protected carriers from smallpox and now reduces the risk for HIV infection. In general, the author’s review of the evidence for and against these hypotheses, which remain speculative, is evenhanded and up-to-date. His accounts of the human and social effects of epidemic diseases and the origins of public health are full of lively anecdotes and colorful detail. Interspersed throughout are personal asides, clinical pearls, and lengthy tutorials on basic science topics, such as DNA replication and gene splicing.

Although this book is far more information dense than are popular books for the lay public, its many shortcomings in terms of organization, depth, and documentation (including surprisingly few references) diminish its value to scholarly readers. More than anything else, it resembles an intellectually inspired but somewhat disorganized professor’s medical school lecture, which would probably be more fun to hear in person than to read. Meanwhile, those who are interested in a 21st-century account of the battle of the genomes may want to wait. Rapid advances in genomic science and technology are opening the way to better understanding of biology, evolution, and medicine, but the full integration of these disciplines is still at a relatively early stage. The idea that genes of 1 species can influence whole ecosystems, described by Richard Dawkins in 1982 as the “extended phenotype” (*2*), is only now giving rise to new perspectives on community genetics (*3*).
